# Non-destructive sampling of larval amphibians shows tail tissues reflect whole-body methylmercury concentrations and trophic-related differences in bioaccumulation

**DOI:** 10.1007/s10646-026-03090-z

**Published:** 2026-07-02

**Authors:** Brian J. Tornabene, Blake R. Hossack, Daniel A. Grear, Thomas L. Anderson, Brad M. Glorioso, J. Hardin Waddle, Jon M. Davenport, Collin A. Eagles-Smith, Caitlin T. Rumrill, Kelly L. Smalling

**Affiliations:** 1 U.S. Geological Survey, Northern Rocky Mountain Science Center, Boise Idaho, 83702 United States; 2 U.S. Geological Survey, Northern Rocky Mountain Science Center, Missoula 59801 Montana, United States; 3 Wildlife Biology Program, University of Montana, Missoula, Montana 59801 United States; 4 U.S. Geological Survey, National Wildlife Health Center, Madison, Wisconsin 53711 United States; 5 Department of Biological Sciences, Southern Illinois University, Box 1651, Edwardsville, Edwardsville, Illinois 62026 United States; 6 U.S. Geological Survey, Wetland and Aquatic Research Center, 70506, Lafayette Louisiana, United States; 7 U.S. Geological Survey, Wetland and Aquatic Research Center, Gainesville, Florida 70506 United States; 8 Department of Biology, Appalachian State University, Boone, North Carolina 28608 United States; 9 U.S. Geological Survey, Forest and Rangeland Ecosystem Science Center, Corvallis, Oregon 97331 United States; 10 U.S. Geological Survey, New Jersey Water Science Center, Lawrenceville, New Jersey 08648 United States

**Keywords:** Anuran, Bioindicator, Caudate, Contaminant, Ecotoxicology, Mercury

## Abstract

Mercury (Hg), and specifically methylmercury (MeHg), is a contaminant of global concern to humans and wildlife, but there is still limited understanding of its effects on many taxa. Amphibians are closely associated with water, where Hg is converted to MeHg, and high concentrations of MeHg can reduce survival of amphibians in the wild. However, limited non-lethal proxies exist for estimating MeHg bioaccumulation in amphibian larvae, which could be used to reduce destructive sampling and allow sampling of imperiled species. We evaluated whether tail clips were indicative of whole-body MeHg concentrations of larval amphibians, whether dragonflies were an effective bioindicator of Hg concentrations of larval amphibians, and compared MeHg bioaccumulation in predatory caudate (salamanders, newts) and non-predatory anuran (frogs, toads) larvae. Tail-clip and whole-body MeHg concentrations were strongly correlated, especially for caudates. There was high variability among sites and species, but caudates had higher MeHg than anurans, and MeHg concentrations of caudates and anurans were strongly correlated. Dragonflies were a weak indicator of MeHg for anurans but strong indicators for larval caudates, likely because caudates and dragonflies are carnivorous and occupy similar trophic positions. Our study revealed that sampling tails can be an effective non-destructive index of whole-body MeHg for larval amphibians and demonstrates differences in MeHg bioaccumulation related to trophic position in larval amphibians and dragonflies. All post-embryo life stages for a broad suite of amphibian species can now be sampled non-lethally for MeHg, allowing for a broader examination of population-level effects and focus on imperiled species that cannot be sampled destructively.

**Supplementary Information:**

The online version contains supplementary material available at https://doi.org/10.1007/s10646-026-03090-z.

## Introduction

Mercury (Hg) is a contaminant of global concern to humans and wildlife (Scheuhammer et al. 2012). Inorganic Hg in the environment can be converted to methylmercury (MeHg) through microbial processes in aquatic ecosystems (Chasar et al. 2009; Driscoll et al. 2013). Methylmercury is more bioavailable and toxic than inorganic Hg, tends to bioaccumulate in local food webs, and can negatively affect terrestrial and aquatic wildlife health (Scheuhammer et al. 2012). In comparison to fish, birds, and mammals, there has been limited research on Hg bioaccumulation for other aquatic and semi-aquatic species such as amphibians, despite their common occurrence in habitats associated with MeHg production (reviewed in Scheuhammer et al. 2007; Chételat et al. 2020).

Amphibians play vital ecological roles, are abundant in many habitats, serve as key components of food webs, and function as bioindicators of environmental change such as increased MeHg (Wells 2010). Based on limited studies, total Hg (THg; inorganic and methylmercury) can reduce adult fecundity and survival of larvae (Unrine et al. 2004; Bergeron et al. 2011a), and MeHg can act independently or through interactions with disease to reduce survival of free-ranging adults (Kain et al. 2025; Tornabene et al. 2025). Despite evidence of the hazards Hg poses to amphibians, studies are still limited primarily to assessments of local contamination in adult life stages (e.g., Tornabene et al. 2023), limiting our understanding of broader exposure and risk to individuals, populations, and ecological communities.

Proxy measurements for estimating contaminants in animals are important tools that can reduce lethal take, especially in small or imperiled populations, and increase sample sizes even for common species. Measurements of Hg and MeHg in abiotic matrices (e.g., water or soil) are generally not effective proxies because physiological and ecological processes can result in inconsistent relationships between abiotic and biological concentrations (Bradford et al. 2023; Tornabene et al. 2023). For many species, blood or other non-lethal tissue samples like fur, scutes, feathers, and even muscle plugs can be used to estimate Hg bioaccumulation (Scheuhammer et al. 2012). However, this can be difficult for small-bodied individuals where handling stress and the need for enough tissue for analysis can pose mortality risks. For adult amphibians, the use of non-lethal tail tips or toe clips has been validated to estimate whole-body Hg concentrations (Tornabene et al. 2023; Rumrill et al. 2026). In contrast, because of their typically smaller size, limited non-lethal proxies exist for estimating Hg concentrations in amphibian larvae (Polich et al. 2013). Amphibian larvae are therefore more likely to be sampled lethally and whole bodies analyzed (e.g., Smalling et al. 2021; Hossack et al. 2025), but results can be biased if larvae are not allowed to purge gut contents prior to analysis limiting comparisons among studies (Burger and Snodgrass 1998, 2001; Smalling et al. 2021). Lethal sampling can also pose challenges when sampling from sensitive populations or protected species, compromising our ability to conduct environmental assessments and measure MeHg bioaccumulation across all life stages and species (Tornabene et al. 2023; Kain et al. 2025).

To facilitate future estimates of Hg risk to larval amphibians, we tested the relationships between MeHg concentrations in non-lethal tissue proxies (tail clips) and whole-body MeHg concentrations to determine their utility for estimating individual-level MeHg bioaccumulation. Tail clipping is often used for genetic sampling in larval salamanders, and effects on fitness are limited (Semlitsch 1990; Wilbur and Semlitsch 1990; Polich et al. 2013). We also tested the relationship between a common site-level bioindicator, dragonfly larvae, and larval amphibians to assess their effectiveness in determining population-level bioaccumulation in amphibians. Dragonflies are effective bioindicators of fish and adult amphibian MeHg bioaccumulation (Eagles-Smith et al. 2020; Tornabene et al. 2023), but larval amphibian proxies are lacking. To do so, we sampled 14 species of frogs and toads (anurans), 5 species of salamanders and newts (caudates), and dragonfly larvae from across the conterminous USA. We assessed correlations between tail-clip concentrations and their paired whole-body concentrations, as well as site-specific paired larval amphibian concentrations and dragonfly concentrations. By sampling anurans and caudates at the same sites, this allowed us to assess variation in bioaccumulation between non-predatory anuran larvae and predatory caudate larvae. Ultimately, greater understanding of variation in MeHg across life histories, phylogenies, and trophic levels can facilitate inferences that are made based on limited sampling and support translating bioaccumulation information into ecological risk.

## Methods

### Site selection and field sampling

**Fig. 1:**
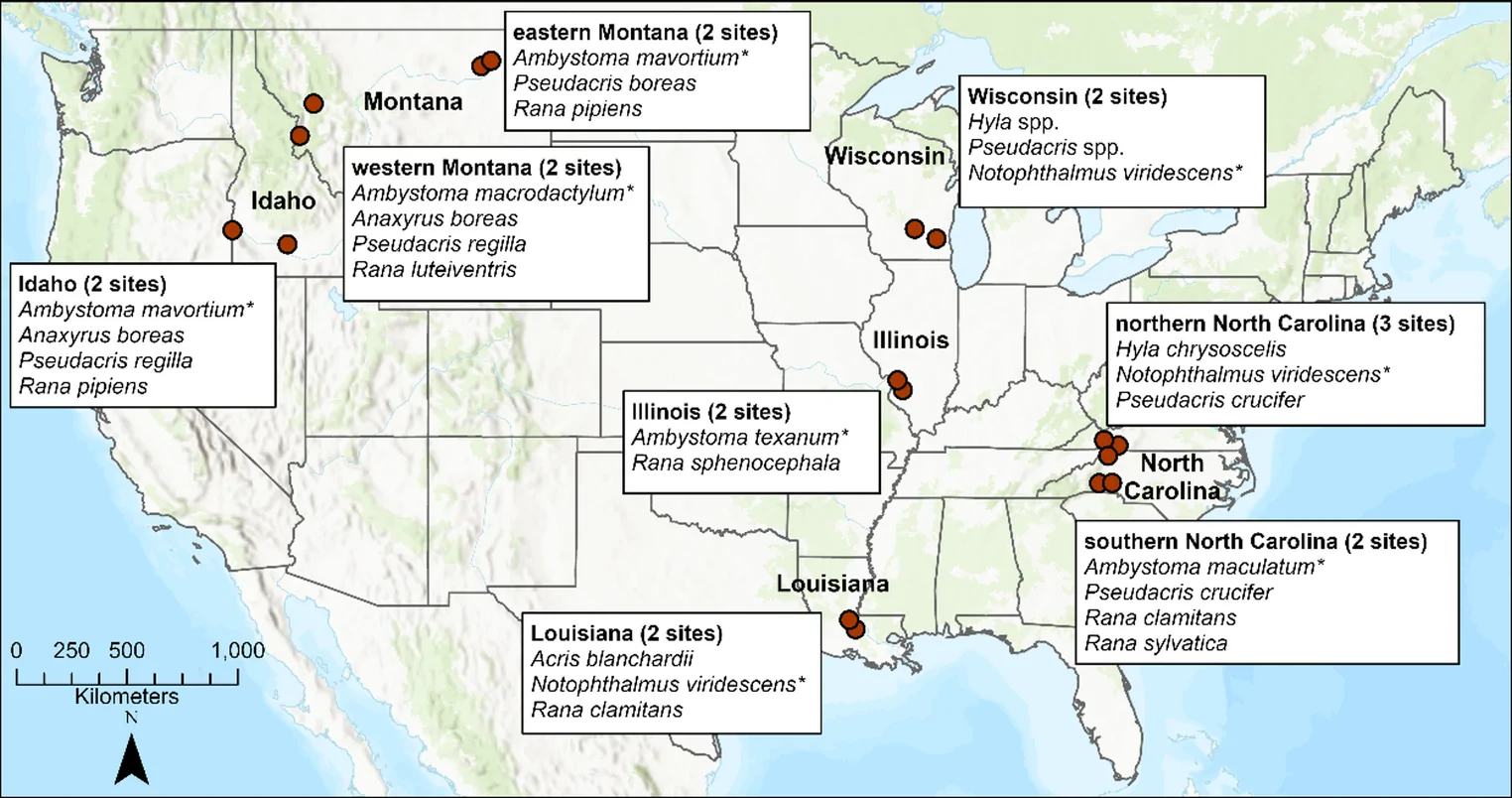
Sites sampled in the conterminous United States to evaluate tail clips as an indicator of whole-body methylmercury, bioaccumulation in amphibians, and correlations with dragonflies at a subset of sites (see Tables S1 and S2). Asterisks (*) next to species names indicate caudates (i.e., salamanders and newts) collected at each site whereas all others are anurans (i.e., frogs and toads). A third site was sampled in Louisiana, but only dragonflies were sampled and included in analyses. Site points are jittered to reduce overlap. Baselayer sources: Esri, Garmin, Food and Agriculture Organization, National Oceanic and Atmospheric Administration, U.S. Geological Survey, Environmental Protection Agency

We sampled 388 individual larval amphibians (255 anurans, 133 caudates) of 19 species from 17 sites in six states throughout the conterminous US in 2023 (Fig. 1). To allow direct comparison of MeHg across life histories, we constrained sampling to sites where we could feasibly collect both anuran and caudate larvae (Smalling et al. 2021). Larval amphibians were collected with dip nets and then held in ample site water for 24 h to allow them to clear their guts (Smalling et al. 2021). Amphibian larvae were identified to species except in Wisconsin where *Hyla* and *Pseudacris* larvae could not be identified beyond genus. Dragonfly larvae were also collected at the same time, at the same sites, identified to family, placed in labeled polyethylene bags, and held on ice until frozen at -20 °C. At one site in Louisiana (BL; not included in Fig. 1), we sampled dragonfly larvae but were unable to collect enough amphibian larvae; these results are only included in dragonfly-specific analyses.

After 24 h, amphibian larvae were euthanized with MS-222 or benzocaine in accordance with U.S. Geological Survey (USGS) or University-specific Institutional Animal Care and Use Committee procedures and then assigned to a development stage (Gosner 1960; Watson and Russell 2000). Individuals were immediately placed in a sealed, labeled polyethylene bag and stored at -20 °C until processing in the laboratory. Frozen samples were shipped to the USGS Contaminant Ecology Laboratory in Corvallis, Oregon, for sample processing and analysis for THg and MeHg concentrations (see below).

### Sample processing and analysis

In the laboratory, snout-vent-length and total length (including the tail) of individual larvae were measured to the nearest mm with a ruler and wet weight was determined to the nearest 0.0001 g on a digital balance. Tail-clip collection followed methods outlined in Pfleeger et al. (2016) and Rumrill et al. (2026). Briefly, the distal 0.5–1 cm of tail was removed depending on length of each larva’s tail to optimize amount of tissue needed for analysis. For a subset of larger Green Frog (*Rana [Lithobates] clamitans*) larvae, we sampled a 1-cm tail clip and analyzed the anterior and posterior halves of that clip individually to better determine the optimal amount of tissue needed to describe relationships with whole-body MeHg and whether tissue location (anterior, posterior, both) mattered. All remaining whole bodies and tail clips were oven-dried (50 °C) to constant mass, and dry weight of respective tissues was determined to the nearest 0.0001 g on a digital balance. Whole bodies and tail clips were homogenized via porcelain mortar and pestle prior to analysis.

Whole bodies and tail clips of amphibians were analyzed for MeHg. A subset of amphibian larvae (see below) and all dragonfly larvae were analyzed for THg. In dragonflies, THg and MeHg are strongly correlated and measuring THg is more cost effective (Eagles-Smith et al. 2020). Methylmercury determination was conducted using a MERX-M automated MeHg analyzer (Brooks Rand Inc, Seattle, Washington, USA) following US EPA Method 1630. THg concentrations were determined at the USGS Contaminant Ecology Research Lab (CERL) using either a Milestone DMA-80 (Milestone Inc., Monroe, Connecticut, USA) or Nippon MA-3000 (Nippon Instrument Corporation, Osaka, Japan) Hg analyzer following EPA method 7473 (EPA 1998). Prior to MeHg analysis, dried samples were digested in 30% nitric acid at 60 °C for 14 h and ethylated with 1% sodium tetraethylborate. Quality-assurance measures included certified reference materials (scallop [IAEA-452; International Atomic Energy Agency, Vienna, Austria] and fish homogenate [IAEA-407; International Atomic Energy Agency, Vienna, Austria]), continuing calibration checks, blanks, matrix spikes, and duplicates. Percent recoveries for certified reference materials and calibration checks averaged (± SE) 99.26% ± 0.27% (*n* = 453) and 100.9% ± 0.3% (*n* = 213), respectively; the relative percent difference for duplicates averaged (± SE) 3.46% ± 0.25% (*n* = 148).

Total Hg determination in dragonfly larvae and a subset of larval amphibian whole bodies was conducted on either a Milestone tri-cell DMA 80 Direct Mercury Analyzer (Milestone Inc, Monroe, Connecticut, USA) or Nippon MA-3000 (Nippon Instruments Corporation, Tokyo, Japan) following US EPA Method 7473 (EPA 1998). We were only able to analyze THg for a subset of larval amphibians for which enough dry mass was available. For these samples, we analyzed THg along with MeHg to evaluate MeHg: THg ratios in amphibians, which can vary based on species, life stage, and trophic position (Tornabene et al. 2023). Quality assurance measures included certified reference materials (lobster hepatopancreas [TORT-3; National Research Council of Canada, Ottawa, Canada] and dogfish muscle [DORM-4; National Research Council of Canada, Ottawa, Canada]), continuing calibration checks, blanks, and duplicates. Percent recoveries for certified reference materials and calibration checks averaged (± SE) 103.8% ± 0.5% (*n* = 51) and 97.75% ± 1.69% (*n* = 51), respectively; the relative percent difference for duplicates averaged (± SE) 4.2% ± 1.2% (*n* = 12).

### Statistical analysis

We used generalized linear mixed models (GLMM; package ‘lme4’; Bates et al. 2014) to analyze the relationship between whole-body (response variable) and tail-clip MeHg concentrations (explanatory variable; ng/g dw). We included amphibian order (caudate or anuran) as an interaction with tail clip (amphibian order × tail-clip concentration) because we expected the relationship between whole-body and tail-clip concentrations could reflect differences in diet. We also included tail-clip length (0.5–1.0 cm) and dry weight of sample (g) as fixed effects to account for the potential influence of variation in these factors and test whether there were differences between 0.5- and 1.0-cm tail clips. We included site and amphibian species as random effects. We calculated both marginal R^2^ (R^2^m), which is variation accounted for by fixed effects, and conditional R^2^ (R^2^c), which is variation accounted for by fixed and random effects (package ‘MuMIn’; Bartón 2010). For the subset of 10 Green Frog larvae where we collected anterior and posterior tail samples, we used ordinary least squares regression to investigate relationships between anterior, posterior, whole-tail (anterior + posterior), and whole-body (anterior + posterior + remainder of body) MeHg concentrations using four separate regressions.

To test the predictive ability of tail tissue to estimate the whole-body MeHg concentrations, we reconstructed whole-body MeHg concentrations using the data available and Eq. 1, in which concentrations are ng/g dw and dry weights are in g. Here, we use ‘reconstructed’ to explain that we mathematically reconstructed whole-body MeHg concentrations from various tissue samples. Whole-tail MeHg concentrations were reconstructed using the data available and Eq. 2, in which concentrations are ng/g dw and dry weights are in g. For the subset of Green Frogs, we used a combination of Eqs. 2 and 1 to get reconstructed whole-body MeHg concentrations. All MeHg concentrations (whole body, tail clip, anterior or posterior tail clip) were natural log transformed to account for skewedness that is common with ecotoxicology data.

1 $$\dfrac{\begin{array}{l}(remainder \, of\, body\, dry\, weight \times\, remainder\, of\, body\, MeHg\, concentration)\\ \qquad +\, (tail\, clip\, dry\, weight \times\, tail\, clip\, MeHg\, concentration)\end{array}}{\begin{array}{l}(remainder\, of\, body\, dry\, weight +\, tail\, clip\, dry\, weight)\end{array}}$$

2 $$\dfrac{\begin{array}{l}(anterior\, tail\, dry\, weight \times\, anterior\, tail\, MeHg\, concentration)\\ \qquad+\, (posterior\, tail\, dry\, weight \times\, posterior\, tail\, MeHg\, concentration)\end{array}}{\begin{array}{l}(anterior\, tail\, dry\, weight + \, posterior\, tail\, dry\, weight)\end{array}}$$

We also evaluated variability in bioaccumulation of whole-body MeHg among different species of larval amphibians as well as relationships with development and animal size. We included site and amphibian species as random effects in all GLMM models (species differences and life history traits). We used three separate GLMMs to evaluate relationships between MeHg concentrations in amphibians and Gosner stage (anurans only), Watson-Russell stage (caudates only), and body weight (dry weight in grams; all species).

Total mercury (THg; methyl and inorganic) concentrations are known to vary consistently among dragonfly families (Eagles-Smith et al. 2020). Therefore, prior to assessing the relationship between amphibian MeHg and dragonfly THg concentrations, we standardized dragonfly THg concentrations to a consistent family (Aeshnidae) by calculating an aeshnid-equivalent concentration (Eagles-Smith et al. 2020). We used a GLMM to evaluate relationships between site-level geometric mean aeshnid-equivalent THg and geometric mean amphibian MeHg at sites where both types of samples were collected. Geometric means were used instead of arithmetic means to account for skewedness that is common in ecotoxicology data. We included both site and amphibian species as random effects. We similarly evaluated correlations among anuran and caudate species MeHg concentrations within sites where both orders of amphibians were collected. We first calculated site-level geometric means separately for each anuran and caudate species at the site level and correlated these means using a GLMM with site as a random effect. All data formatting, reconstructing of concentrations, and analyses were conducted in program R using RStudio (Posit team 2025; R Core Team 2025). All assumptions for the statistical analyses (e.g., linearity, independence, homoscedasticity, normality of residuals) described above were evaluated in program R and met.

## Results

**Fig. 2:**
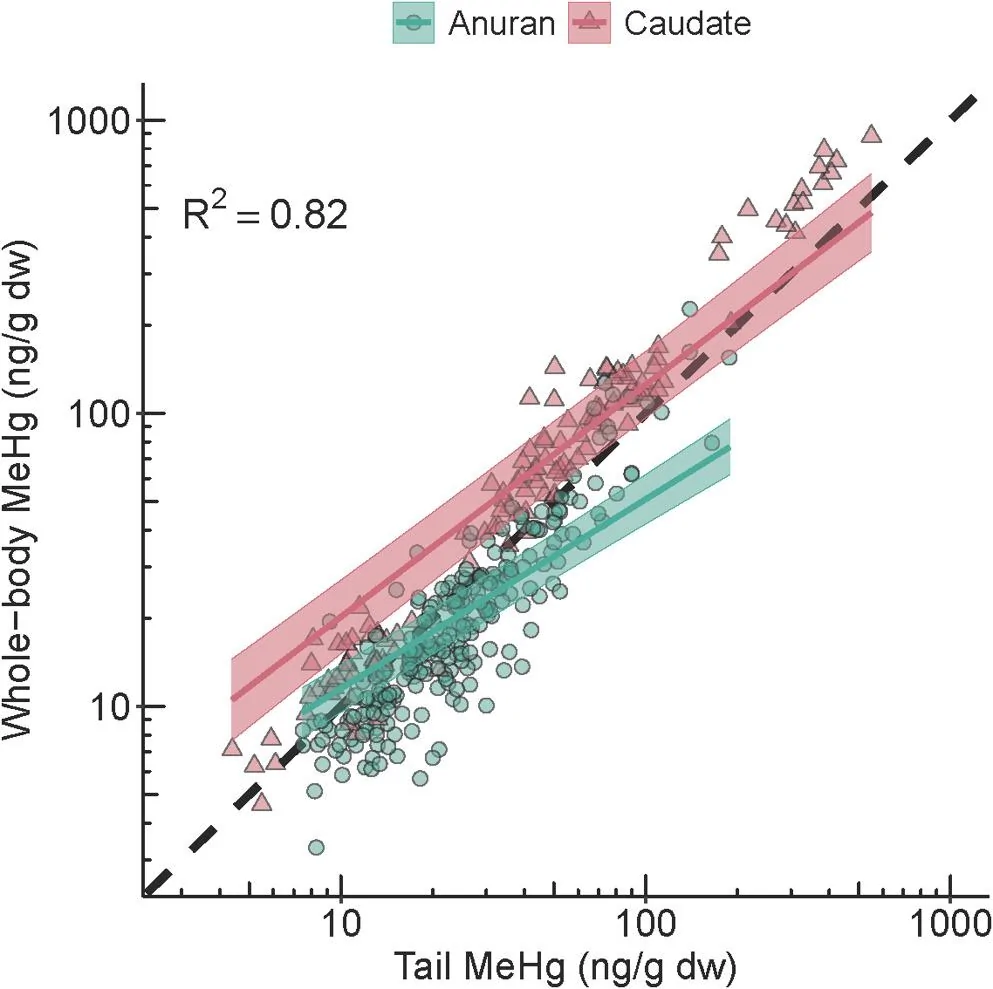
Relationship (mean (± 95% confidence interval) between whole-body (tail clip + remainder of whole body) and tail-clip methylmercury (MeHg) concentrations (ng/g dry weight [dw]). Whole-body MeHg was reconstructed from tail and remainder of body that were analyzed separately for MeHg of larval anurans and caudates. The red line and triangles denote caudates (salamanders and newts), and the green line and circles denote anurans (frogs and toads). The dashed line represents a 1:1 relationship between whole-body and tail MeHg

Methylmercury concentrations in tail clips and reconstructed whole bodies of larval amphibians were strongly correlated across all 19 species (R^2^ = 0.82; Fig. 2 and Table S1 and Equations S1 and S2). However, the relationship between tail-clip and whole-body MeHg concentrations differed between amphibian orders. Caudates generally had consistently higher concentrations in both tissue types than anurans; slopes differed statistically but the differences were not particularly strong (Fig. 2 and Table S3). Methylmercury concentrations in caudate whole bodies and tails were also more strongly correlated with each other (R^2^ = 0.93) than in anurans (R^2^ = 0.45). Neither tail-clip length nor dry weight influenced the relationship between tail-clip and whole-body concentrations (Table S1). Whereas the fixed effects accounted for most of the variation in the data ([fixed effects] R^2^m = 0.82), the random effects accounted for additional variation ([fixed and random effects] R^2^c = 0.96). Mean MeHg: THg ratios, for the subset of samples analyzed for THg (*n* = 50), in whole bodies was 69.8% for caudates and anurans combined (standard deviation [SD] = 26.4%; range = 17.9–111%). Mean MeHg: THg ratios in whole bodies were higher in caudates (85.70%; SD = 11.7%) than in anurans (46.0%; SD = 24.4%).

Relationships varied among anterior tail, posterior tail, reconstructed whole-tail (anterior + posterior), and reconstructed whole-body MeHg concentrations (posterior + anterior + remainder of body) based on the subset of 10 Green Frog larvae (Figure S1 and Table S2 and Equations S3–6). Whole-tail MeHg concentrations were more strongly correlated with whole-body concentrations than either tail section (R^2^ = 0.86; Figure S1D and Table S4). Anterior and posterior tail clips each explained substantial variation in whole-body MeHg (R^2^ = 0.76 and 0.67; Figure S1B and C), even though the anterior and posterior tail-clip MeHg concentrations were less strongly correlated with each other (R^2^ = 0.45; Figure S1A).

**Fig. 3:**
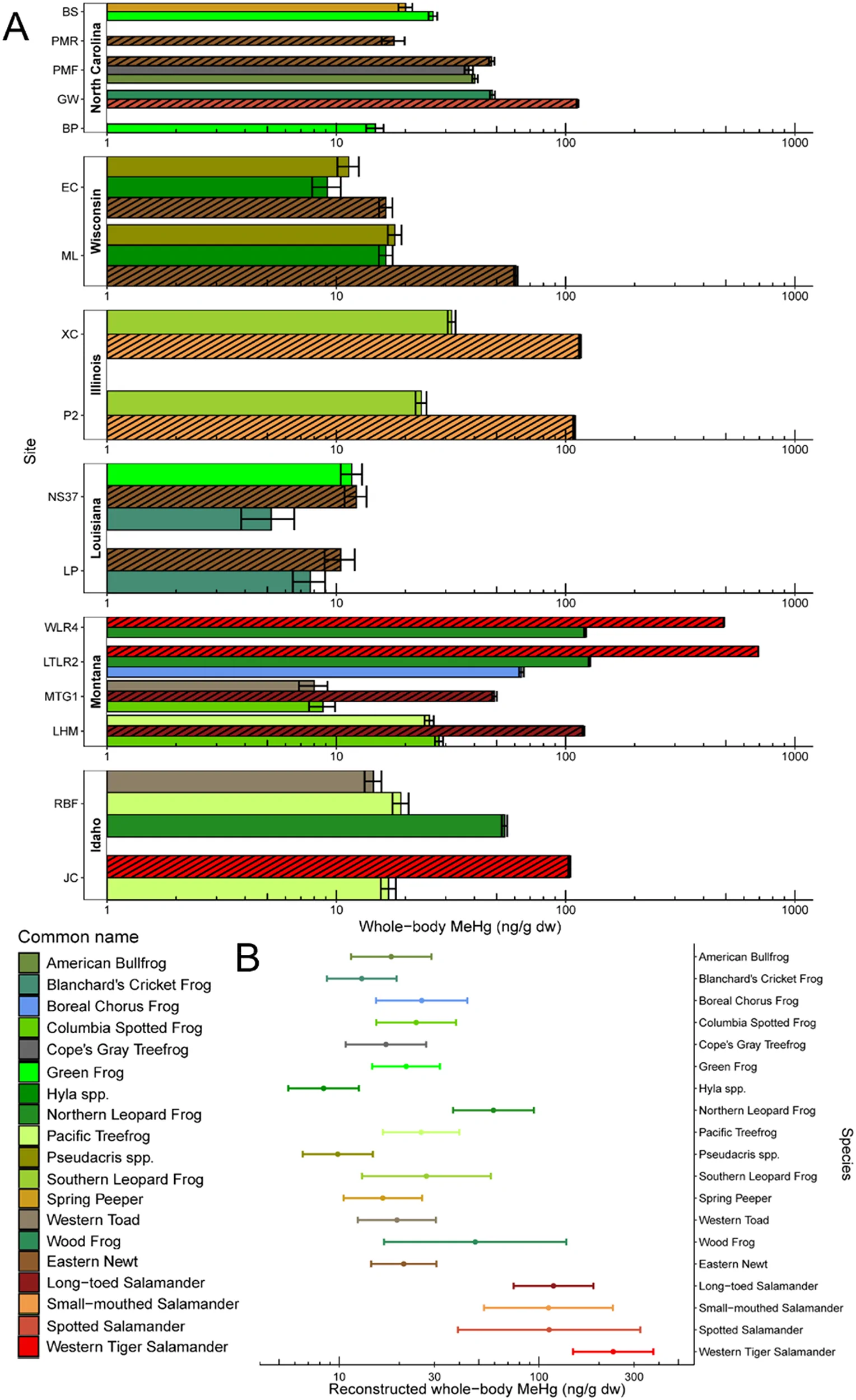
(**A**) Geometric mean (± geometric standard deviation) whole-body methylmercury (MeHg; ng/g dry weight [dw]) concentrations of larval amphibians sampled across the contiguous United States. Bars for caudates (salamanders and newts) are crosshatched for comparison with anurans in solid colors (**B**) Model-estimated mean (and 95% confidence intervals) whole-body methylmercury (tail clip + remainder of whole body; MeHg; ng/g dw) for larvae of each species. Whole-body MeHg was reconstructed from tail and remainder of body that were analyzed separately for MeHg

Methylmercury concentrations varied widely among larval amphibian taxa, though species were partially confounded with sites because most species were sampled at only one site (Fig. 3 and S2 and Table S3). There was a 133-fold difference between sites with the highest (693 ng/g dw MeHg; Western Tiger Salamanders in Montana [*Ambystoma mavortium*; site LTLR2]) and lowest geometric mean MeHg concentrations (5.2 ng/g dw MeHg; Blanchard’s Cricket Frog [*Acris blanchardi*] in Louisiana [NS37]). Within amphibian orders, anurans had a 24-fold difference among species with the highest (127 ng/g dw MeHg Northern Leopard Frogs [*R. pipiens*] in Montana [LTLR2]) and lowest (5.2 ng/g dw MeHg; Blanchard’s Cricket Frog in Louisiana [NS37]) geometric mean MeHg concentrations. There was a 66-fold difference among caudates (693 ng/g dw MeHg; Western Tiger Salamanders in Montana [LTLR2] and 10.5 ng/g dw MeHg; Eastern Newts in Louisiana [*Notophthalmus viridescens*; LP]). Eastern Newts were sampled across the largest geographic range (six individual sites across Wisconsin, Louisiana, and North Carolina), and there was a 5.8-fold difference between minimum and maximum geometric mean MeHg concentrations. Western Tiger Salamanders had the highest estimated mean MeHg, followed by Spotted, Small-mouthed, and Long-toed salamanders (Fig. 3 and Table S4). Anurans generally had lower model-estimated mean MeHg, with *Hyla* spp. having the lowest estimated MeHg.

Based on caudate and anuran samples taken from the same sites (14 sites), caudates had higher geometric MeHg concentrations than anurans at 13 of 14 (93%) sites; only Eastern Newts in North Carolina had lower geometric mean MeHg concentrations (23.9 ng/g dw) than Green Frogs (26.6 ng/g dw), but this was not the case between these species and Spring Peepers (*Pseudacris crucifer*; 21.1 ng/g dw; Fig. 3). Geometric mean MeHg concentrations for caudates (geometric mean = 149 ng/g dw MeHg) were 3.16 times higher (95% confidence interval 2.32‒4.30 times higher) than anurans (geometric mean = 33.0 ng/g dw MeHg; Figure S3]). However, MeHg concentrations of anurans and caudates were strongly correlated with each other at sites where both were collected (R^2^m = 0.74, R^2^c = 0.90; Figure S4 and Equation S7).

Whole-body MeHg concentrations were unrelated to developmental stage for anurans or caudates (Figure S5 and Table S5). However, the relationship between developmental stage and MeHg concentrations varied widely among species and populations (Figures S6 and S7). There was clear evidence that the relationship between dry weight of larval amphibians and MeHg concentrations depended upon amphibian order (some support for an interaction term). Whereas MeHg concentrations slightly increased with dry weight for caudates, concentrations decreased with dry weight for anurans (Figure S8 and Table S5). The relationship between dry weight and MeHg concentrations also varied widely among species and sites with some showing a positive, negative, or no relationship among the two variables (Figure S9).

**Fig. 4:**
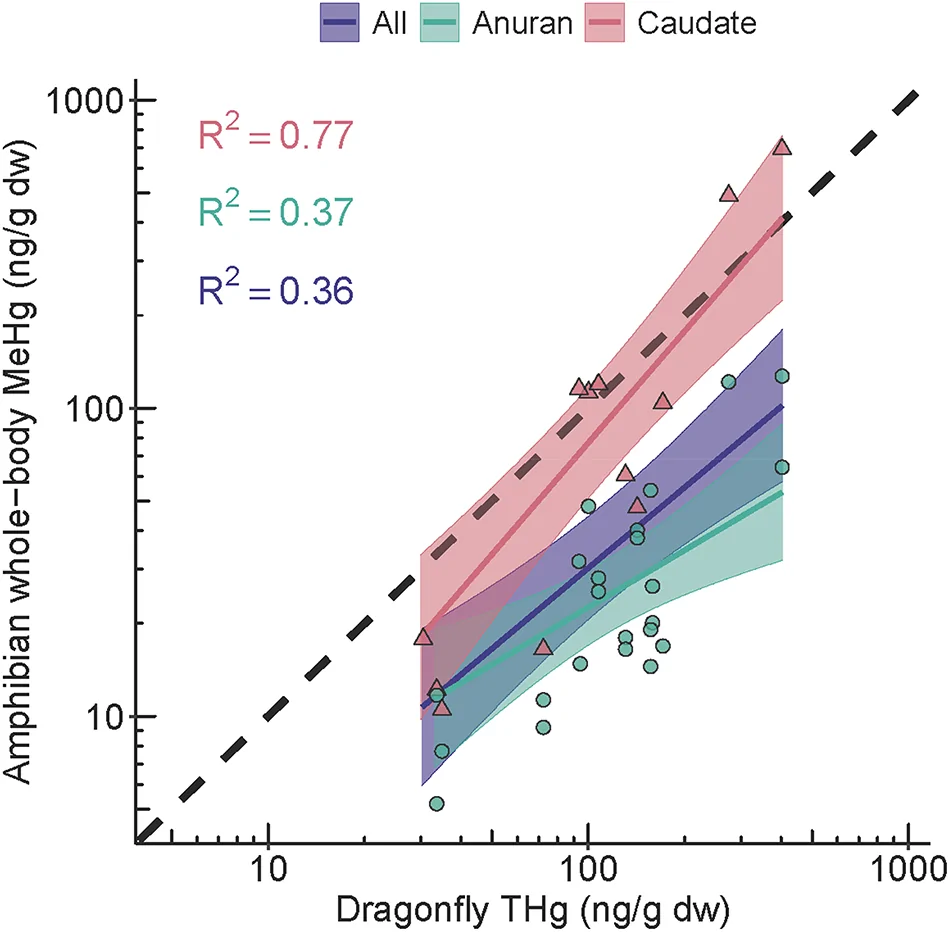
Relationships between paired geometric mean (± 95% confidence interval) larval amphibian whole-body methylmercury (MeHg; ng/g dry weight [dw]) and dragonfly larvae (Aeshnid-equivalent) total mercury (THg, ng/g dw) concentrations from the same locations across the conterminous United States. Whole-body larval MeHg was reconstructed from tail and remainder of body samples that were analyzed separately. Triangles denote caudates (salamanders and newts) and circles denote anurans (frogs and toads). The red line shows the estimate for caudates, the green line for anurans, and the blue line for all amphibians combined

At the site level, geometric mean MeHg concentrations for larval amphibians were correlated with geometric mean THg concentrations for larval dragonflies, but the relationships differed between anurans and caudates (Figs. 4 and S10 and Tables S6 and S7). The strongest correlation was between dragonflies and caudates (R^2^ = 0.77), whereas anuran and pooled relationships were much weaker (R^2^ = 0.37 and 0.36, respectively; Equations S8–10). In dragonflies, there was a 13-fold difference between sites with the highest (LTLR2 in Montana; geometric mean = 403 ng/g dw THg) and lowest geometric mean THg concentrations (PMR in North Carolina; geometric mean = 30.5 ng/g dw THg).

## Discussion

We assessed the utility of non-lethal sampling by comparing MeHg concentrations in tails to whole bodies for 19 species of larval amphibians. Previous studies have determined that non-lethal sampling of toes or tails is indicative of whole-body MeHg concentrations of juvenile and adult amphibians (Pfleeger et al. 2016; Tornabene et al. 2023; Rumrill et al. 2026), but non-lethal sampling had not been evaluated for larvae. We found MeHg concentration in tails strongly correlated with whole-body MeHg concentrations, but the portion of tail sampled could affect results. Caudate tails were also more strongly related to whole-body MeHg concentrations compared to anurans, perhaps because caudates generally have larger, more muscular tails (or longer tail to body ratios; Duellman and Trueb 1994).

Our results indicated measuring MeHg in the ‘whole tail’ (1 cm; sometimes but not always whole tail of samples) of larvae was most indicative of whole-body MeHg concentrations, but a 0.5-cm sample from the posterior of the tail was still strongly related to whole-body MeHg concentrations. We chose 0.5-cm tail clips to ensure we had enough tissue for analysis, but also because we thought 0.5 cm would limit potential for mortality. If tissue collection is likely to cause mortality, there would be little reason not to use whole bodies. In the wild, amphibian larvae often lose portions of their tails, presumably from failed predation attempts (Nunes et al. 2010). The consequences of tail tissue loss are not well documented, but experiments based on tail clipping have reported either no fitness-associated consequences for anuran or caudate larvae (up to 75% of tail; Wilbur and Semlitsch 1990; up to 1 cm of tail; Polich et al. 2013) or reduced survival and behavioral changes in larvae with substantial tail clips (up to 75% tail removal; Semlitsch 1990; Nunes et al. 2010). Nevertheless, most amphibian larvae can quickly regenerate their tails (e.g., in < 15 days; Nunes et al. 2010), which could help offset effects of tail samples (Wilbur and Semlitsch 1990). It was unexpected that anterior and posterior tail clips were not more strongly correlated and that their relationships with whole-body MeHg concentrations differed. We suspect this could reflect differences in amount of muscle tissue between the two segments of tail given that MeHg binds to amino acids and proteins more than other tissues (Mason et al. 1995). Regardless, these results suggest that sampling 0.5 cm of the posterior portion of the tail would allow reasonably precise estimates of whole-body MeHg while limiting injury to larvae.

The wide variation in larval amphibian MeHg concentrations among sites and species (133-fold difference among sites) exceeded that of a previous nationwide study on adult amphibians (33-fold among sites; Tornabene et al. 2023), likely because we sampled across a larger diversity and size range of amphibians. Some concentrations rivaled those of larger, adult amphibians with the highest MeHg concentrations in the previous study (geometric mean = 1,071 ng/g dw in adult Gulf Coast Waterdogs [*Necturus beyeri*] in Louisiana compared to 693 ng/g dw in larval Western Tiger Salamanders [*Ambystoma mavortium*] in Montana; Tornabene et al. 2023). Although all larval amphibians sampled had MeHg concentrations below impairment benchmarks for fishes (~ 800 ng/g dw; Ahmed et al. 2022; Tornabene et al. 2023), a recent study highlighted that 164 ng/g dw MeHg could reduce adult amphibian survival by 20% and 469 ng/g dw MeHg could reduce survival by 55% (Tornabene et al. 2025). Only 5% of our samples had concentrations above 164 ng/g dw and only 3% had concentrations above 469 ng/g dw, but studies evaluating effects of MeHg bioaccumulation on field-collected larval amphibians and all other life stages remain rare, limiting our ability to infer effects from concentrations (Tornabene et al. 2023). From studies in sites contaminated by coal ash residues, which can contain other heavy metals and contaminants, MeHg can have detrimental effects on behavior, growth, metamorphosis, and survival of larvae, particularly when larvae come from mothers with elevated Hg concentrations (e.g., Bergeron et al. 2011b; Todd et al. 2011).

Caudates generally had higher MeHg concentrations than anurans from the same sites. We expected this relationship because predatory larval caudates occupy higher trophic levels than non-predatory anurans and are therefore more likely to bioaccumulate MeHg, but previous studies based on THg have not supported this expectation (Bergeron et al. 2010; Smalling et al. 2021). To our knowledge, ours is the only study to compare MeHg bioaccumulation in larval caudates and anurans from the same sites, so we do not know if comparisons based on THg produce similar results as those based on MeHg. The notable exception to the pattern of caudates having higher MeHg concentrations than anurans was for Eastern Newts (*Notophthalmus viridescens)*. The comparatively low concentration of MeHg in Eastern Newts matches results from a previous study based on sampling adults, which we suspect may result from the unusual life history of newts (e.g., long periods as terrestrial efts before metamorphosing again into aquatic adults), or that they are generally smaller than other salamander larvae we sampled and thus may occupy lower trophic levels (Petranka 1998; Tornabene et al. 2023).

Dragonfly THg concentrations were similarly correlated with caudate MeHg, when collected at the same site (R^2^ = 0.77), as correlations with adult amphibian MeHg in a previous study where few caudates were sampled (Tornabene et al. 2023; R^2^ = 0.76 and 0.67 for all species and ranids in previous study). Methylmercury of larval caudates had a stronger correlation with dragonfly THg collected from the same site, compared to larval anurans (R^2^ = 0.37), perhaps because both caudates and dragonflies occupy similar trophic levels as aquatic predators, as described above. The strong correlation between caudate and anuran MeHg concentrations across species within the same sites (R^2^ = 0.74), and the apparently minor changes associated with larval development, may facilitate making community-level inferences without having to sample all species. However, it is still unclear whether dragonflies are strong proxies for larval anurans that occupy lower trophic levels.

By sampling across a wide diversity of amphibian species and using multiple cross-sections of tails, we determined that 0.5-cm samples from tails can be used as a nondestructive index of whole-body MeHg concentrations in larval amphibians limiting or precluding larval mortality. Nevertheless, there could be some mass limitations for small species or samples with smaller tails (e.g., below detection limits) and this could be addressed on a species and sample basis. The ability to use tissue samples to measure MeHg offers several advantages compared to collecting whole bodies, including reducing impacts on survival and populations, allowing larger sample sizes, and not having to hold individuals to allow them to clear their gut content that could bias results. This method might also be useful to understand bioaccumulation of other contaminants (e.g., selenium, cyanotoxins, or PFAS) in larval amphibians. All post-embryo life stages of amphibians (larvae, juveniles, and adults) can now be sampled non-lethally for MeHg, dependent on size and MeHg content (e.g., above detection limits), allowing for a broader examination of population-level effects and a focus on imperiled species that cannot be sampled destructively. Future studies applying non-destructive sampling may help clarify how MeHg, and perhaps other contaminants, bioaccumulate in amphibians occupying different habitats and how readily MeHg is transferred from the aquatic to terrestrial environments when larvae metamorphose (Hossack et al. 2025). A greater understanding of variation in MeHg across life histories, phylogenies, and trophic levels could strengthen inferences based on limited sampling and facilitate translating bioaccumulation information into ecological risk.

## Supplementary Information

Below is the link to the electronic supplementary material.

Supplementary Material 1

## Data Availability

Data are available at https://doi.org/10.5066/P16HVH7D.
