# Supplementary material for: Non-destructive sampling of larval amphibians shows tail tissues reflect whole-body methylmercury concentrations and trophic-related differences in bioaccumulation
Source: Ecotoxicology. 2026 Jul 2;35(6):138. doi: 10.1007/s10646-026-03090-z (PMC13328247; doi:10.1007/s10646-026-03090-z)
Supplement: Supplementary file 1 — Supplementary Material 1 [file 10646_2026_3090_MOESM1_ESM.pdf]

Supplementary for:

Non-destructive sampling of larval amphibians shows tail tissues reflect whole-body methylmercury concentrations and trophic-related differences in bioaccumulation

Brian J. Tornabene[1], Blake R. Hossack[2,3], Daniel A. Grear[4], Thomas L. Anderson[5], Brad M. Glorioso[6], J. Hardin Waddle[7], Jon M. Davenport[8], Collin A. Eagles-Smith[9], Caitlin T. Rumrill[9], and Kelly L. Smalling[10]

[1]U.S. Geological Survey, Northern Rocky Mountain Science Center, Boise, Idaho 83702, United States
[2]U.S. Geological Survey, Northern Rocky Mountain Science Center, Missoula, Montana 59801, United States
[3]Wildlife Biology Program, University of Montana, Missoula, Montana 59801, United States
[4]U.S. Geological Survey, National Wildlife Health Center, Madison, Wisconsin 53711, United States
[5]Department of Biological Sciences, Southern Illinois University Edwardsville, Box 1651, Edwardsville, Illinois 62026, United States
[6]U.S. Geological Survey, Wetland and Aquatic Research Center, Lafayette, Louisiana 70506, United States
[7]U.S. Geological Survey, Wetland and Aquatic Research Center, Gainesville, Florida 32653, United States
[8]Department of Biology, Appalachian State University, Boone, North Carolina 28608, United States
[9]U.S. Geological Survey, Forest and Rangeland Ecosystem Science Center, Corvallis, Oregon 97331, United States
[10]U.S. Geological Survey, New Jersey Water Science Center, Lawrenceville, New Jersey 08648, United States
*Corresponding author: orcid.org/0000-0002-2348-3119; Email: btornabene@usgs.gov

Number of:

Tables: 7
Figures: 10
Equations: 10

Supplementary Tables

Table S1. Summary statistics for mixed-effects regression model evaluating relationships between reconstructed whole-body and tail-clip methylmercury (MeHg; ng/g dry weight; log-transformed) in amphibians across the conterminous United States. Whole-body MeHg was reconstructed from tail and remainder of body that were analyzed separately for MeHg. SE = standard error. The reference level for tail clip length is 0.5 cm and the reference level for amphibian order is anurans. *p*-values < 0.10 are **bolded** for reference.

Variable	Estimate	SE	t	*p*
Intercept	0.95	0.15	6.34	**< 0.001**
Tail MeHg	0.65	0.04	18.03	**< 0.001**
Order: Caudate	0.23	0.23	1.01	0.318
Tail-clip length: 1 cm	0.02	0.03	0.58	0.563
Dry weight (g)	0.03	0.06	0.50	0.616
Tail MeHg × Order: Caudate	0.15	0.05	3.16	**0.002**

Table S2. Summary statistics for linear regressions evaluating relationships among anterior tail clips, posterior tail clips, whole-tail clips (reconstructed from anterior + posterior tail clips) and reconstructed whole-body (posterior + anterior + body remainder) methylmercury (MeHg; ng/g dry weight; log-transformed) of Green Frogs (*Rana clamitans*; n = 10) collected from two different sites in Louisiana and North Carolina, USA. All variables were natural log transformed. SE = standard error. *p*-values < 0.10 are **bolded** for reference.

Response	Explanatory	Estimate	SE	t	*p*	R^2
Anterior	Intercept	1.17	0.58	2.02	**0.078**	0.446
	Posterior	0.58	0.20	2.87	**0.021**	
Whole-body	Intercept	0.38	0.40	0.96	0.363	0.762
	Anterior	0.77	0.14	5.45	**0.001**	
Whole-body	Intercept	0.86	0.38	2.23	**0.056**	0.671
	Posterior	0.59	0.13	4.40	**0.002**	
Whole-body	Intercept	0.23	0.31	0.72	0.491	0.858
	Whole-tail	0.82	0.11	7.45	**< 0.001**	

Table S3. Summary statistics for whole-body methylmercury (MeHg; ng/g dry weight; log-transformed) from larval amphibian sampled at sites across the contiguous United States. Whole-body MeHg was reconstructed from tail and remainder of body that were analyzed separately for MeHg. SD = standard deviation.

Site ID	State	Common name	Order	Count	Geometric mean	Geometric SD	Minimum	Maximum	Latitude	Longitude
BS	North Carolina	Green Frog	anuran	10	26.40	1.13	20.79	33.46	36.2293	-81.6414
BS	North Carolina	Spring Peeper	anuran	10	20.06	1.38	12.61	41.24	36.2293	-81.6414
PMR	North Carolina	Eastern Newt	caudate	10	17.82	2.05	11.04	70.08	36.5645	-81.6429
PMF	North Carolina	American Bullfrog	anuran	6	40.22	1.15	33.51	47.79	36.5674	-81.6458
PMF	North Carolina	Cope's Gray Treefrog	anuran	6	37.85	1.62	21.63	79.28	36.5674	-81.6458
PMF	North Carolina	Eastern Newt	caudate	10	47.56	1.31	31.10	64.64	36.5674	-81.6458
GW	North Carolina	Spotted Salamander	caudate	10	112.58	1.21	75.99	153.49	36.2075	-81.6515
GW	North Carolina	Wood Frog	anuran	10	47.99	1.18	36.26	61.26	36.2075	-81.6515
BP	North Carolina	Green Frog	anuran	10	14.79	1.29	9.82	24.23	36.2105	-81.6933
EC	Wisconsin	*Pseudacris* spp..	anuran	11	11.33	1.22	8.22	15.35	42.8710	-88.5472
EC	Wisconsin	Eastern Newt	caudate	10	16.45	1.10	14.05	18.78	42.8710	-88.5472
EC	Wisconsin	*Hyla* spp.	anuran	9	9.15	1.31	5.83	13.45	42.8710	-88.5472
ML	Wisconsin	*Pseudacris* spp.	anuran	10	18.00	1.23	14.02	24.23	43.419	-89.2897
ML	Wisconsin	Eastern Newt	caudate	9	60.63	1.12	50.64	69.76	43.419	-89.2897
ML	Wisconsin	*Hyla* spp.	anuran	10	16.46	1.15	13.46	20.56	43.419	-89.2897
XC	Illinois	Small-mouthed Salamander	caudate	10	115.51	1.26	79.87	144.82	38.8056	-89.9889
XC	Illinois	Southern Leopard Frog	anuran	10	31.84	1.28	23.45	44.31	38.8056	-89.9889
P2	Illinois	Small-mouthed Salamander	caudate	10	108.93	1.30	74.98	143.51	38.7976	-90.0082
P2	Illinois	Southern Leopard Frog	anuran	10	23.46	1.28	18.79	38.97	38.7976	-90.0082
NS37	Louisiana	Blanchard's Cricket Frog	anuran	4	5.20	1.36	3.30	6.37	30.4237	-91.6453
NS37	Louisiana	Eastern Newt	caudate	13	12.21	1.34	8.08	21.83	30.4237	-91.6453
NS37	Louisiana	Green Frog	anuran	20	11.69	1.23	7.99	15.96	30.4237	-91.6453
LP	Louisiana	Blanchard's Cricket Frog	anuran	16	7.70	1.24	5.14	11.56	30.4249	-91.6639
LP	Louisiana	Eastern Newt	caudate	13	10.45	1.57	4.64	19.45	30.4249	-91.6639
WLR4	Montana	Northern Leopard Frog	anuran	6	121.58	1.47	82.56	226.87	48.0257	-106.6775
WLR4	Montana	Western Tiger Salamander	caudate	10	489.72	1.25	349.85	692.41	48.0257	-106.6775

LTLR2	Montana	Boreal Chorus Frog	anuran	6	64.28	1.53	38.73	108.54	47.9481	-106.905
LTLR2	Montana	Northern Leopard Frog	anuran	4	126.99	1.15	113.98	154.91	47.9481	-106.905
LTLR2	Montana	Western Tiger Salamander	caudate	5	692.99	1.24	516.82	882.81	47.9481	-106.905
MTG1	Montana	Columbia Spotted Frog	anuran	10	8.74	1.13	7.39	10.88	46.9964	-113.7021
MTG1	Montana	Long-toed Salamander	caudate	11	48.95	1.17	38.96	61.85	46.9964	-113.7021
MTG1	Montana	Western Toad	anuran	9	8.01	1.14	6.34	9.64	46.9964	-113.7021
LHM	Montana	Columbia Spotted Frog	anuran	11	28.05	1.15	22.98	36.27	46.0971	-114.264
LHM	Montana	Long-toed Salamander	caudate	10	119.43	1.31	83.95	203.03	46.0971	-114.264
LHM	Montana	Pacific Tree Frog	anuran	9	25.44	1.16	21.33	32.58	46.0971	-114.264
RBF	Idaho	Northern Leopard Frog	anuran	3	54.15	1.5	40.09	85.75	42.9651	-114.7642
RBF	Idaho	Pacific Tree Frog	anuran	14	19.11	1.54	10.67	53.26	42.9651	-114.7642
RBF	Idaho	Western Toad	anuran	16	14.51	1.22	12.01	24.23	42.9651	-114.7642
JC	Idaho	Pacific Tree Frog	anuran	15	16.89	1.27	10.68	25.48	43.3847	-117.002
JC	Idaho	Western Tiger Salamander	caudate	3	103.88	1.07	96.57	110.95	43.3847	-117.002

Table S4. Summary statistics from mixed-effects regression models evaluating differences in accumulation of whole-body methylmercury (MeHg; ng/g dry weight; log-transformed) in different species of larval amphibians collected from the conterminous United States. SE = standard error. Whole-body MeHg was reconstructed from tail and remainder of body that were analyzed separately for MeHg. The model was fit without an intercept such that estimates are species means and SE. *p*-values < 0.10 are **bolded** for reference.

Variable	Estimate	SE	*t*	*p*
Blanchard's Cricket Frog	2.56	0.20	12.51	**< 0.001**
Boreal Chorus Frog	3.25	0.27	2.05	**0.049**
American Bullfrog	2.90	0.24	2.00	**0.047**
Pseudacris spp.	2.29	0.21	-2.21	**0.028**
Columbia Spotted Frog	3.19	0.23	2.02	**0.056**
Cope's Gray Treefrog	2.84	0.24	1.64	0.102
Eastern Newt	3.05	0.19	5.49	**< 0.001**
Green Frog	3.07	0.20	4.50	**< 0.001**
Long-toed Salamander	4.77	0.23	7.11	**< 0.001**
Northern Leopard Frog	4.08	0.24	4.85	**< 0.001**
Pacific Treefrog	3.25	0.22	2.25	**0.036**
Small-mouthed Salamander	4.72	0.38	5.01	**< 0.001**
Southern Leopard Frog	3.31	0.38	1.73	0.101
Spotted Salamander	4.72	0.53	3.77	**0.002**
Spring Peeper	2.81	0.23	1.44	0.151
Hyla spp.	2.12	0.21	-3.47	**< 0.001**
Western Tiger Salamander	5.46	0.24	9.29	**< 0.001**
Western Toad	2.97	0.23	1.32	0.201
Wood Frog	3.87	0.53	2.29	**0.036**

Table S5. Summary statistics from mixed-effects regression models evaluating relationships among whole-body methylmercury accumulation (ng/g dry weight; log-transformed) and Gosner stage (anurans only), Watson-Russell stage (caudates only), and dry weight (g; all species of amphibians) from the conterminous United States. Whole-body MeHg was reconstructed from tail and remainder of body that were analyzed separately for MeHg. The reference level for amphibian order is anurans. Marginal R^2 (R^2m) represents variance explained by fixed effects in models whereas the conditional R^2 (R^2c) represents variance explained by both fixed and random (site and amphibian species) effects in models. *p*-values < 0.10 are **bolded** for reference.

**Model**	**Variable**	**Estimate**	**SE**	***t***	***p***	**R^2m**	**R^2c**
Gosner stage	Intercept	2.72	0.23	11.61	**< 0.001**	0.006	0.879
	Gosner stage	0.01	0.004	2.41	**0.017**		
Watson-Russell stage	Intercept	4.79	1.07	4.46	**< 0.001**	0.0004	0.926
	Watson-Russell stage	-0.02	0.06	-0.31	0.757		
Dry weight	Intercept	2.92	0.22	13.17	**< 0.001**	0.334	0.925
	Dry weight	-0.05	0.03	-1.43	0.154		
	Order: Caudate	1.55	0.27	5.68	**< 0.001**		
	Dry weight × Order: Caudate	0.10	0.06	1.79	**0.075**		

Table S6. Summary statistics for aeshnid-equivalent THg (ng/g dry weight; after standardizing THg calculations among different dragonfly families using published equations) from sites across the conterminous United States where larval amphibians were also collected. SD = standard deviation. Note that larval dragonflies were collected at BL in Louisiana, but not larval amphibians, and are not included in analyses with amphibians (only analyses comparing larval dragonflies).

Site ID	State	Families	Count	Geometric mean	Geometric SD	Minimum	Maximum	Latitude	Longitude
BS	North Carolina	Aeshnidae, Corduliidae, Libellulidae	15	158.70	1.50	59.00	279.00	36.2293	-81.6414
PMR	North Carolina	Aeshnidae	12	30.50	1.20	22.00	41.00	36.5645	-81.6429
PMF	North Carolina	Aeshnidae	12	142.20	1.20	112.00	220.00	36.5674	-81.6458
GW	North Carolina	Aeshnidae, Libellulidae	26	100.10	1.80	40.22	300.00	36.2075	-81.6515
BP	North Carolina	Aeshnidae, Libellulidae	19	94.40	1.70	30.59	224.00	36.2105	-81.6933
EC	Wisconsin	Corduliidae, Libellulidae	12	72.40	1.10	56.70	85.18	44.8771	-88.5457
ML	Wisconsin	Aeshnidae, Libellulidae	4	130.70	1.10	115.00	149.83	43.4190	-89.2897
BL	Louisiana	Aeshnidae, Gomphidae, Macromiidae	16	216.20	1.50	92.97	490.54	30.3650	-89.9225
XC	Illinois	Libellulidae	15	93.50	1.20	70.63	115.29	38.8056	-89.9889
NS37	Louisiana	Libellulidae	15	33.60	1.20	25.08	47.52	30.4237	-91.6453
LP	Louisiana	Libellulidae	14	34.90	1.30	20.57	52.64	30.4249	-91.6639
WLR4	Montana	Aeshnidae	6	274.50	1.40	183.00	484.00	48.0257	-106.6775
LTLR2	Montana	Aeshnidae, Libellulidae	15	403.10	1.20	292.00	608.00	47.9481	-106.9050
MTG1	Montana	Libellulidae	17	44.80	1.20	30.59	53.66	46.9964	-113.7021
LHM	Montana	Corduliidae, Libellulidae	15	107.60	1.10	73.57	127.24	46.0971	-114.2640
RBF	Idaho	Aeshnidae, Corduliidae, Libellulidae	15	156.50	1.20	102.26	219.00	42.9651	-114.7642
JC	Idaho	Aeshnidae, Libellulidae	15	170.90	1.30	64.70	213.66	43.3847	-117.0020

Table S7. Summary statistics for mixed-effects regression models evaluating relationships between mean whole-body methylmercury (MeHg; ng/g dry weight; log-transformed) and aeshnid-equivalent total mercury (THg; ng/g dw; log-transformed; after standardizing THg calculations among different dragonfly families using published equations) from amphibians and dragonflies collected from the same locations. Whole-body MeHg was reconstructed from tail and remainder of body that were analyzed separately for MeHg. The 'Amphibian order' model assesses whether the relationship between amphibian and dragonfly mercury is dependent on amphibian order (anuran = frogs and toads or caudate = salamanders and newts). The reference level for amphibian order is 'anurans'. Marginal R^2 (R^2m) represents variance explained by fixed effects in models whereas the conditional R^2 (R^2c) represents variance explained by both fixed and random (site and amphibian species) effects in models. *p*-values < 0.10 are **bolded** for reference.

Model	Variable	Estimate	SE	*t*	*p*	R^2m	R^2c
All	Intercept	-0.60	0.80	-0.75	0.467	0.364	0.922
	Dragonfly THg	0.87	0.16	5.31	**< 0.001**		
Amphibian order	Intercept	0.28	0.81	0.34	0.737	0.725	0.967
	Dragonfly THg	0.62	0.17	3.67	**0.001**		
	Order: Caudate	-1.47	0.88	-1.67	0.110		
	Dragonfly THg × Order: Caudate	0.59	0.18	3.27	**0.004**		

Supplementary Figures

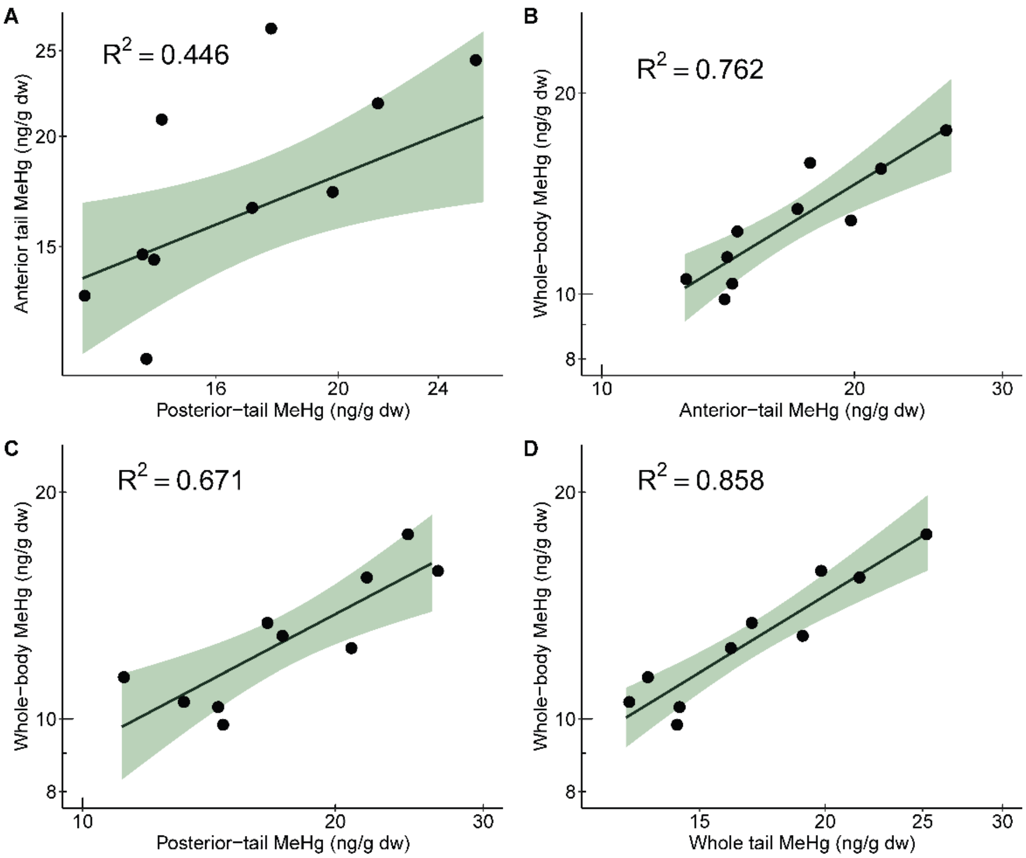

Figure S1. Relationships among mean (± 95% confidence interval) anterior clip, posterior tail clip, whole tail clip (reconstructed from anterior + posterior tail clips) and reconstructed whole body (posterior + anterior + body remainder) methylmercury concentrations (MeHg; ng/g dry weight) of Green Frogs (*Rana clamitans*; n = 10) collected from two different sites in Louisiana and North Carolina, USA.

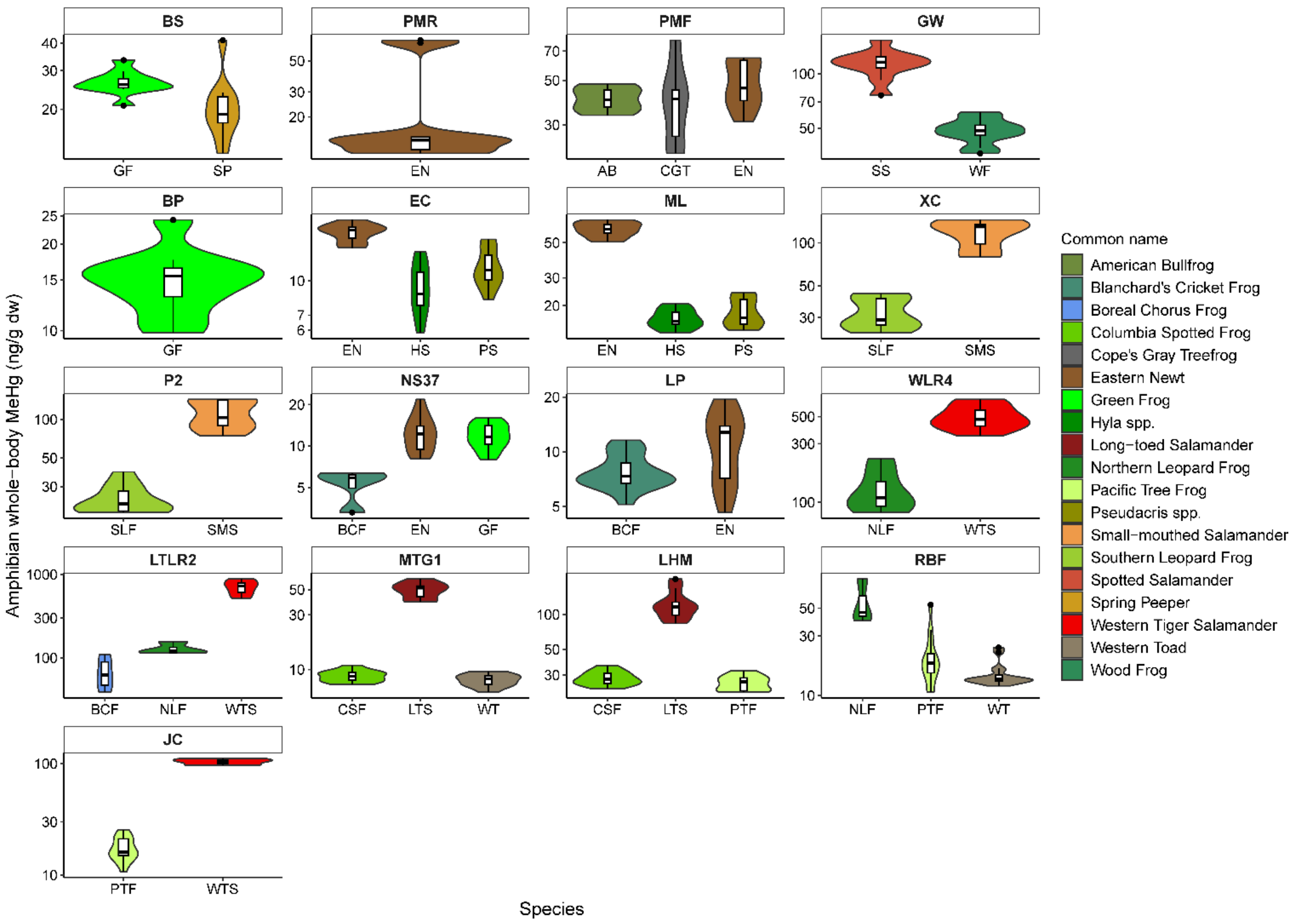

Figure S2. Variation in larval amphibian whole-body methylmercury (MeHg; ng/g dry weight [dw]) bioaccumulation among species and sites represented with violin and boxplots. Note different scales of y-axis in each facet. Species are described by color and generalized acronyms for common names used on the x-axis are spelled out in the legend.

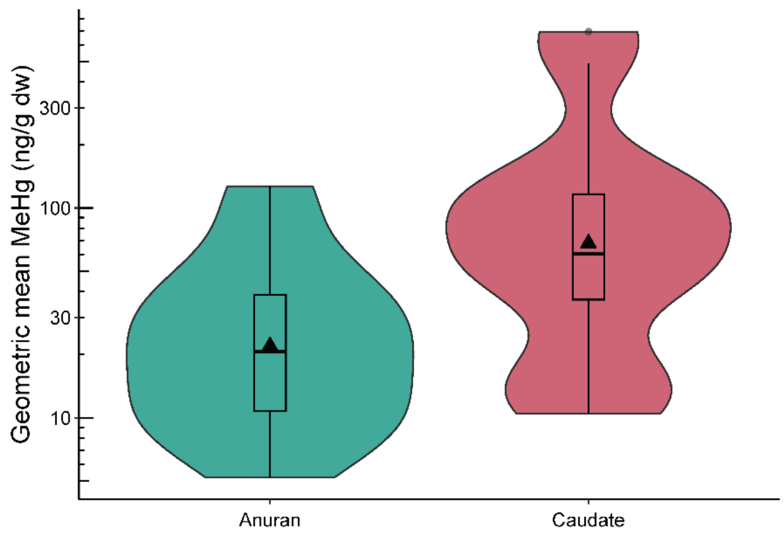

Figure S3. Violin and boxplots of median (horizontal line in boxplot) and geometric mean (triangle) methylmercury (MeHg; ng/g dry weight [dw]) of anurans and caudates collected at the same sites. Caudate MeHg was 3.16× higher than anuran (95% confidence interval: 2.32-4.30× higher; t = -7.85, *p* = < 0.001).

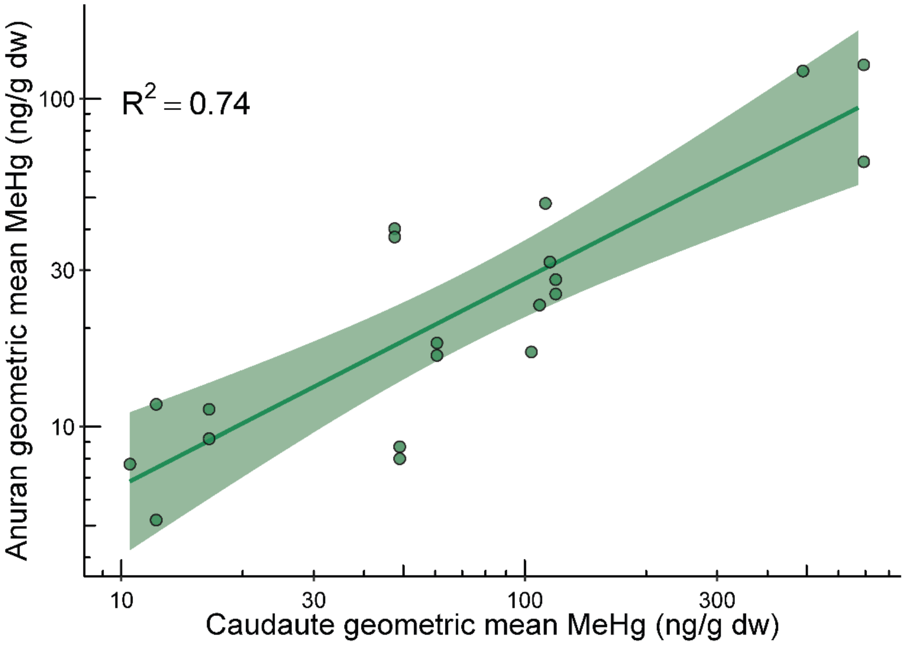

Figure S4. Relationship between mean (± 95% confidence interval) whole-body methylmercury (MeHg; ng/g dw) in anurans and caudates sampled from the same location at 13 sites across the conterminous United States. Whole body MeHg was reconstructed from tail clips and remainder of body that were analyzed separately. Methylmercury concentrations of anurans were strongly correlated with those of caudates (GLMM with site as a random effect; $t = 7.25$; $p < 0.001$).

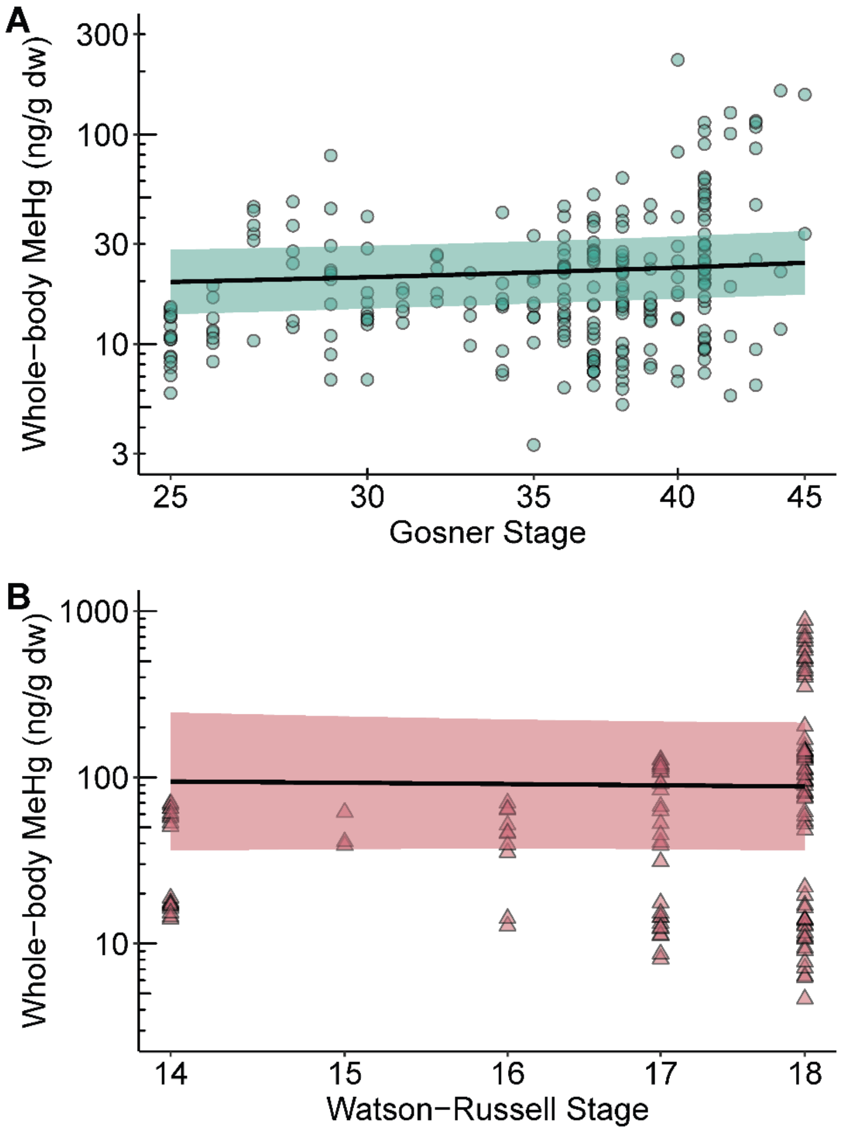

Figure S5. Relationships between mean Gosner (A; anurans only) or Watson-Russell stage (B; caudates only) and reconstructed whole-body methylmercury (MeHg; ng/g dry weight [dw]). Whole-body MeHg was reconstructed from tail and remainder of body that were analyzed separately for MeHg. See Table S5 for summary statistics.

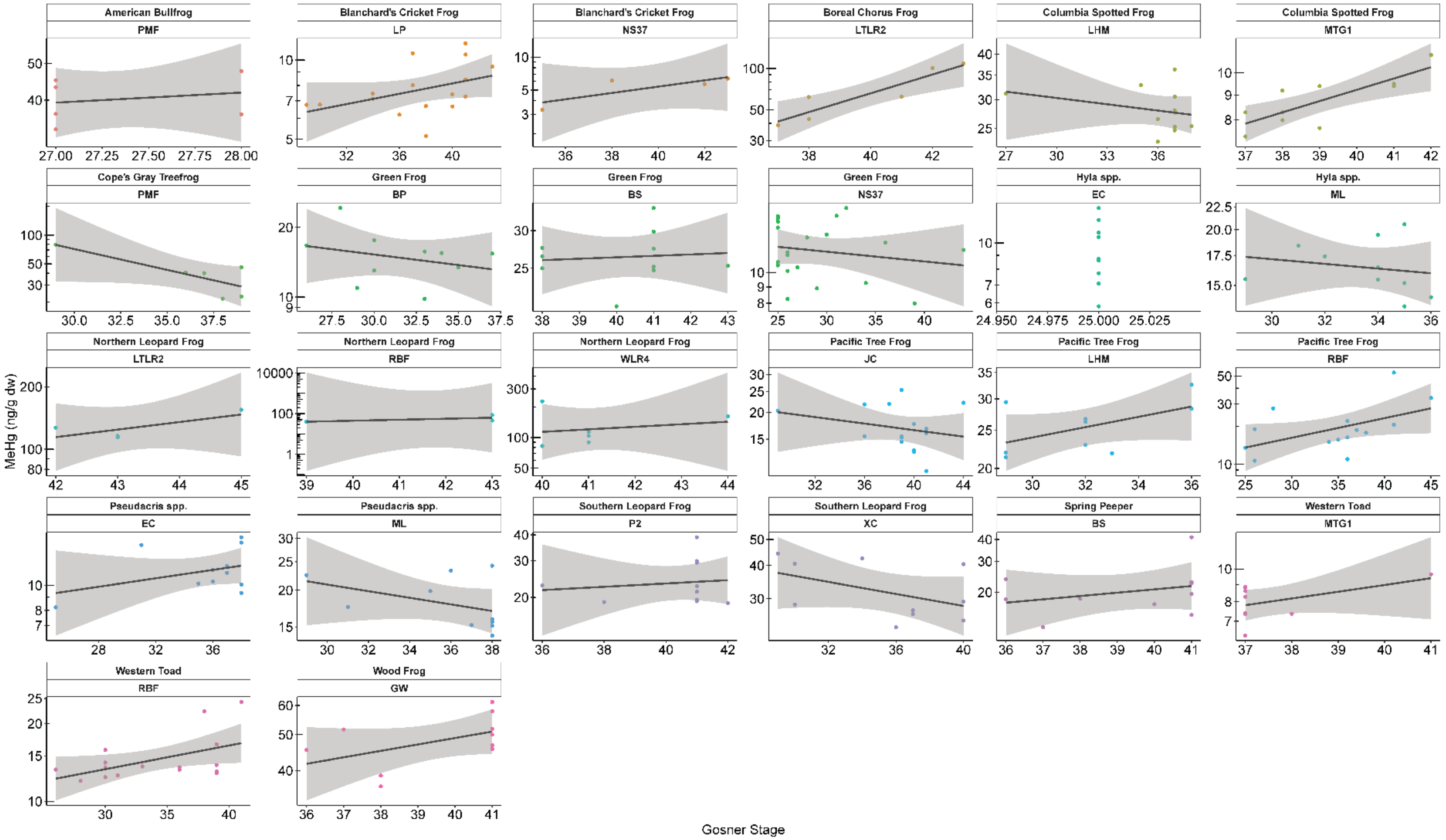

Figure S6. Variation in the relationship between Gosner stage and methylmercury (MeHg; ng/g dry weight [dw]) among species and sites for larval anurans collected from across the conterminous USA. Whole-body MeHg was reconstructed from tail and remainder of body that were analyzed separately for MeHg. For some site-species combinations, only a single life history stage was collected so no relationship is shown.

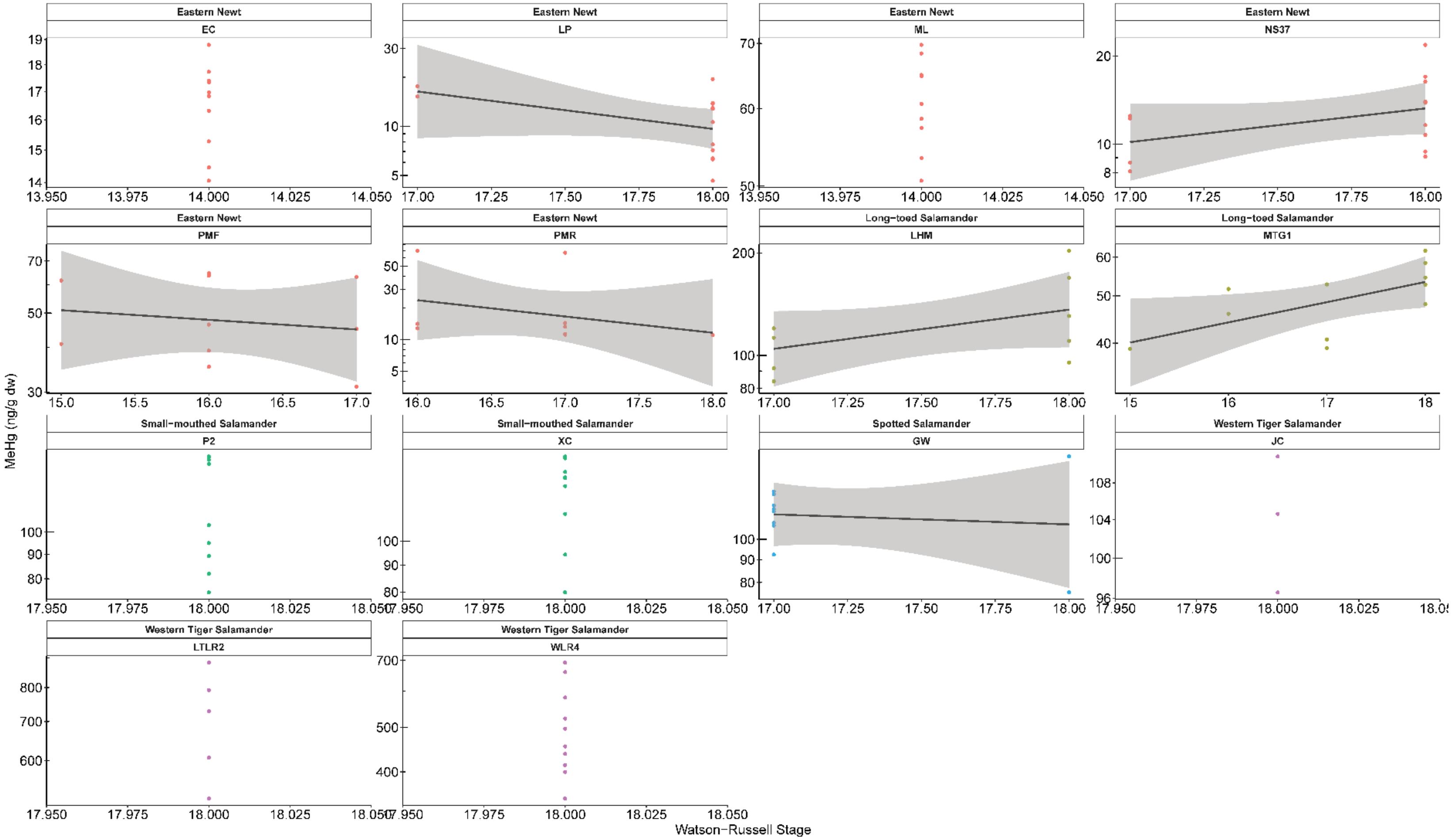

Figure S7. Variation in the relationship between Watson-Russell stage and methylmercury (MeHg; ng/g dry weight [dw]) among species and sites for larval caudates collected from across the conterminous USA. Whole-body MeHg was reconstructed from tail and remainder of body that were analyzed separately for MeHg. For some site-species combinations, only a single life history stage was collected so no relationship is shown.

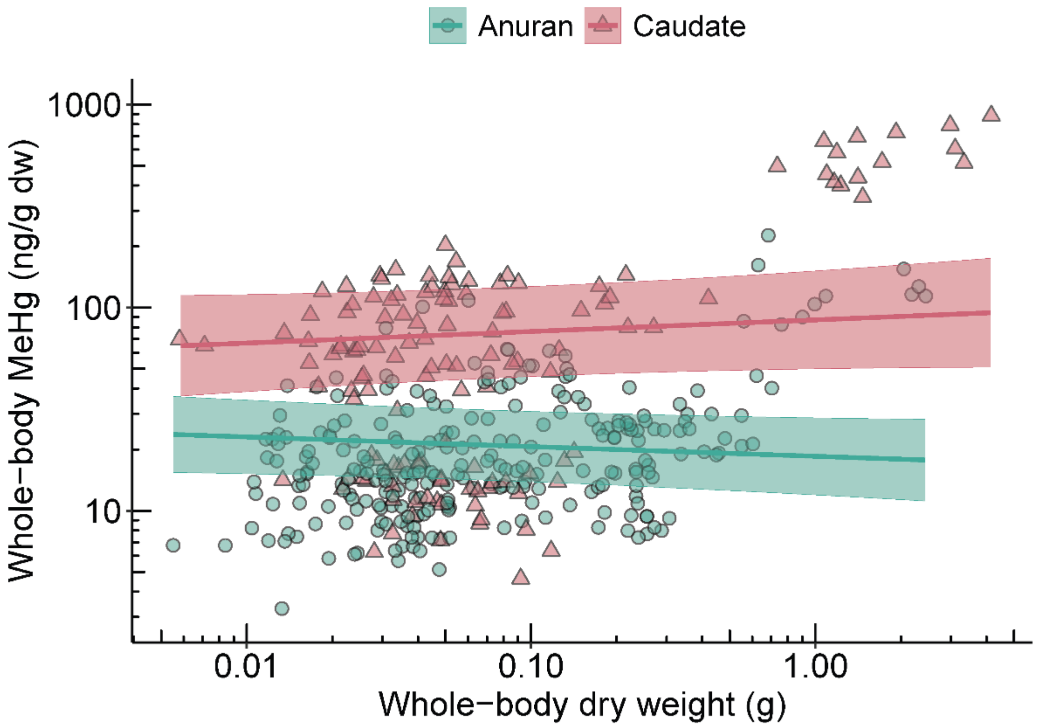

Figure S8. Relationships between mean (± 95% confidence interval) dry weight (g) and reconstructed whole-body MeHg (ng/g dry weight [dw]) by order (anuran or caudate). Whole-body MeHg was reconstructed from tail and remainder of body that were analyzed separately for MeHg. See Table S5 for summary statistics.

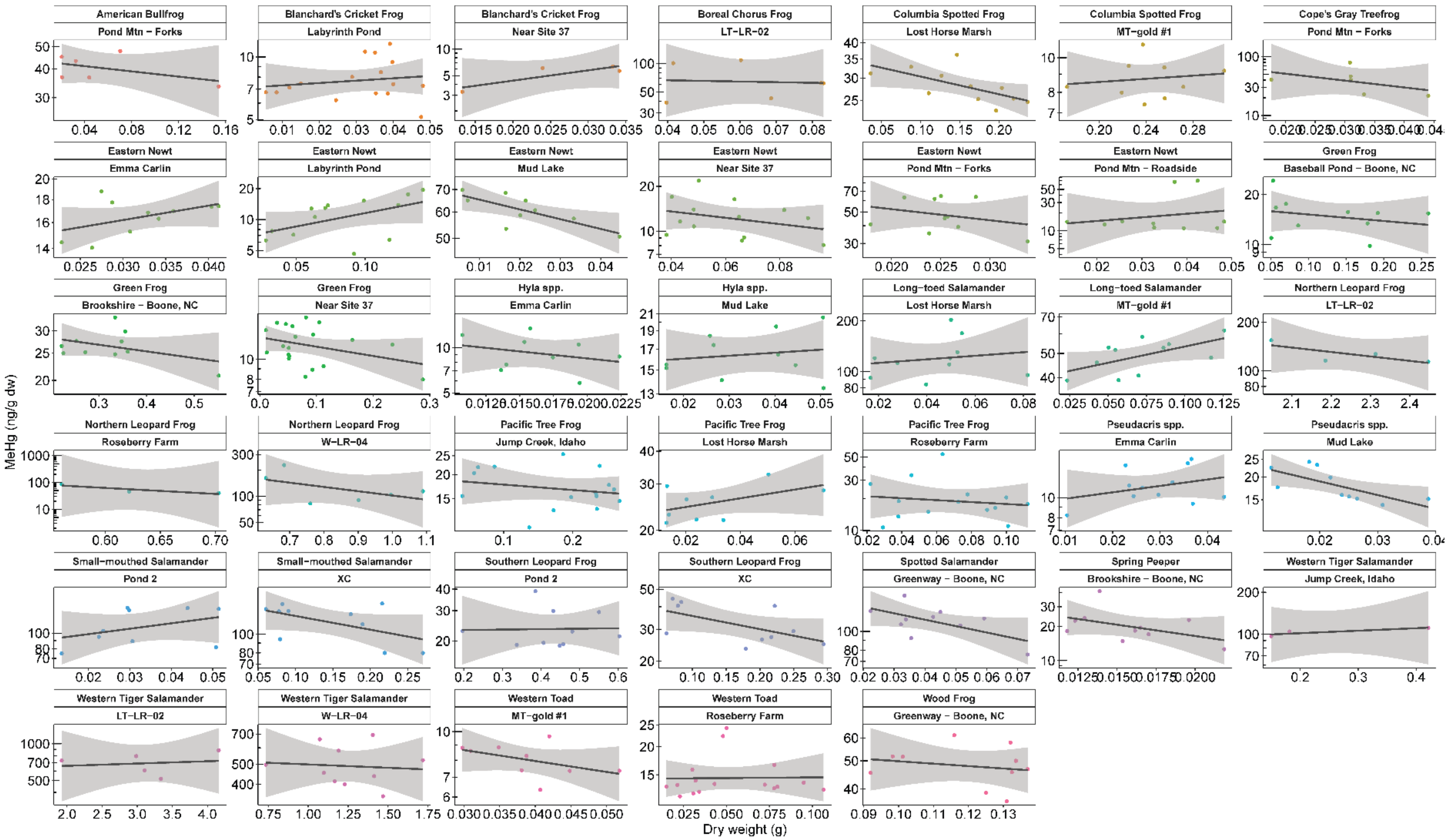

Figure S9. Variation in the relationship between dry weight (g) and methylmercury (MeHg; ng/g dry weight [dw]) among species and sites for larval amphibians collected from across the conterminous USA. Whole-body MeHg was reconstructed from tail and remainder of body that were analyzed separately for MeHg.

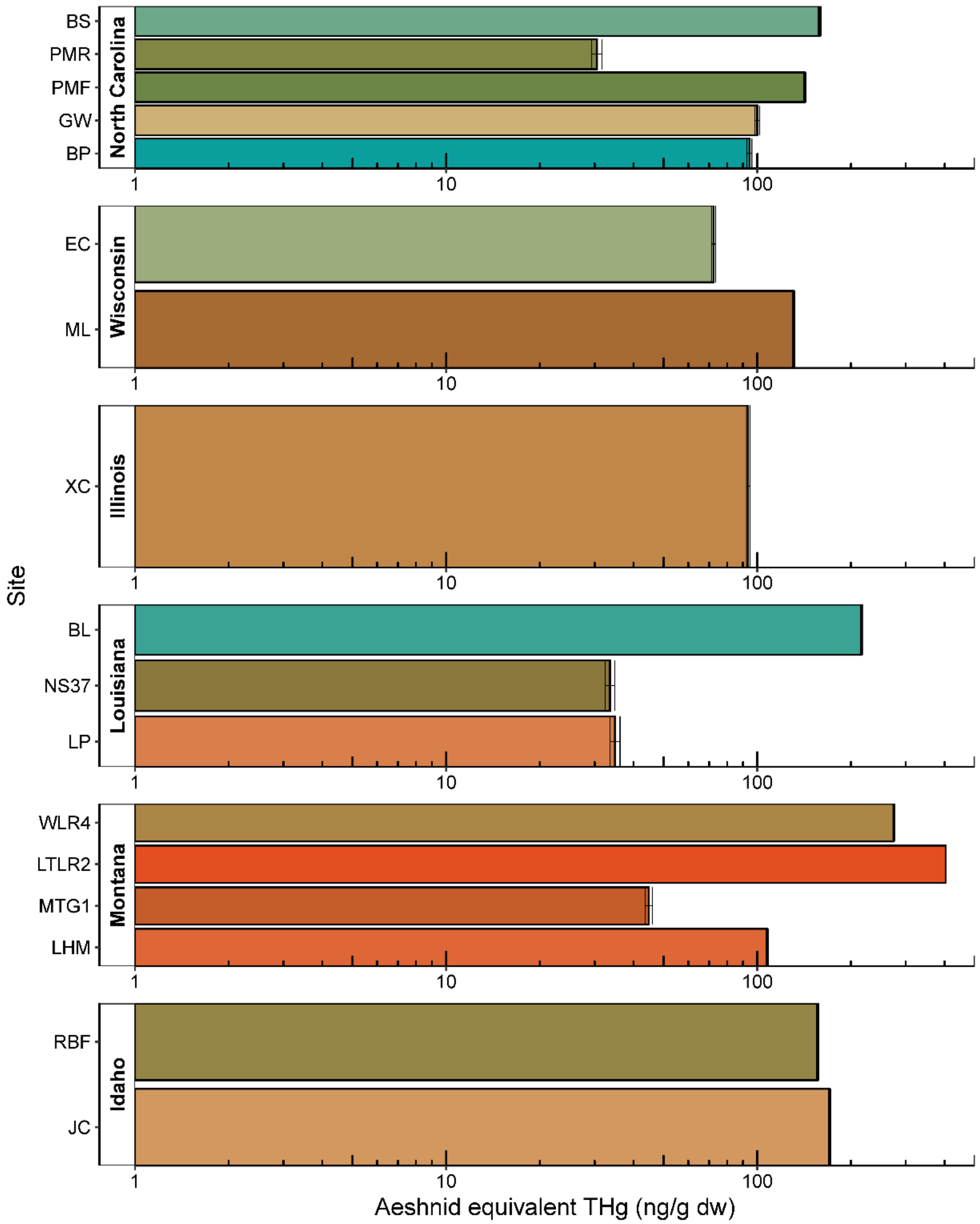

Figure S10. Geometric mean (with geometric standard deviation) Aeshnid-equivalent total mercury (THg; ng/g dry weight [dw]; after standardizing THg calculations among different dragonfly families using published equations) of dragonfly larvae collected from across the conterminous United States where amphibian larvae were also collected. One exception is that amphibian larvae samples from BL in Louisiana could not be run and only dragonfly data are displayed here for comparison to other sites.

Supplementary Equations

Equations S1 and S2: Regression equations describing relationship between reconstructed whole-body (tail clip + remainder of whole body) and tail-clip methylmercury concentrations (MeHg; ng/g dry weight [dw]) of larval amphibians sampled across the United States (N = 389) from mixed-effects linear models with fixed effects of tail MeHg concentrations (separate equations for anurans[S1] and caudates [S2]) and random effects accounting for variation among sites and species. These equations are standalone and from subsetted data (anuran and caudate) simplified from the full model (including all data and predictors; see main text) and do not include terms for tail-clip length and dry weight included in the full model that were non-statistically significant.

$$\boldsymbol{S1})\ ln \text{ anuran whole-body MeHg } \left(\frac{\text{ng}}{\text{g}}\text{dw}\right) = 1.068 + 0.613 \times ln \text{ anuran tail MeHg} \left(\frac{\text{ng}}{\text{g}}\text{dw}\right); R^2m = 0.450, R^2c = 0.898$$

$$\boldsymbol{S2})\ ln \text{ caudate whole-body MeHg } \left(\frac{\text{ng}}{\text{g}}\text{dw}\right) = 0.657 + 0.928 \times ln \text{ caudate tail MeHg} \left(\frac{\text{ng}}{\text{g}}\text{dw}\right);\ R^2m = 0.934, R^2c = 0.972$$

Equation S3: Regression equation describing relationship between anterior- and posterior-tail methylmercury concentrations (MeHg; ng/g dry weight [dw]) of Green Frogs (*Rana clamitans*; n = 10) collected from two different sites in Louisiana and North Carolina, USA.

$$ln \text{ anterior-tail MeHg } \left(\frac{\text{ng}}{\text{g}}\text{dw}\right) = 1.166 + 0.577 \times ln \text{ posterior-tail MeHg} \left(\frac{\text{ng}}{\text{g}}\text{dw}\right);\ R^2 = 0.446$$

Equation S4: Regression equation describing relationship between reconstructed-whole-body (posterior + anterior + body remainder) and anterior-tail methylmercury concentrations (MeHg; ng/g dry weight [dw]) of Green Frogs (*Rana clamitans*; n = 10) collected from two different sites in Louisiana and North Carolina, USA.

$$ln \text{ whole-body MeHg } \left(\frac{\text{ng}}{\text{g}}\text{dw}\right) = 0.383 + 0.767 \times ln \text{ anterior-tail MeHg} \left(\frac{\text{ng}}{\text{g}}\text{dw}\right);\ R^2 = 0.762$$

Equation S5: Regression equation describing relationship between reconstructed-whole-body (posterior + anterior + body remainder) and posterior-tail methylmercury concentrations (MeHg; ng/g dry weight [dw]) of Green Frogs (*Rana clamitans*; n = 10) collected from two different sites in Louisiana and North Carolina, USA.

$$ln \text{ whole-body MeHg } \left(\frac{\text{ng}}{\text{g}}\text{dw}\right) = 0.859 + 0.589 \times ln\, p\text{osterior-tail MeHg} \left(\frac{\text{ng}}{\text{g}}\text{dw}\right);\ R^2 = 0.671$$

Equation S6: Regression equation describing relationship between reconstructed-whole-body (posterior + anterior + body remainder) and reconstructed whole-tail (anterior + posterior) methylmercury concentrations (MeHg; ng/g dry weight [dw]) of Green Frogs (*Rana clamitans*; n = 10) collected from two different sites in Louisiana and North Carolina, USA.

$$ln \text{ whole-body MeHg } \left(\frac{\text{ng}}{\text{g}}\text{dw}\right) = 0.225 + 0.819 \times ln \text{ whole-tail MeHg} \left(\frac{\text{ng}}{\text{g}}\text{dw}\right);\ R^2 = 0.858$$

Equation S7: Regression equation describing relationship between reconstructed whole-body MeHg (ng/g dry weight [dw]) in caudates compared to anurans at sites where they were both

collected. The mixed linear regression model also included a random effect to account for variation among sites. The marginal and conditional R-squared values from the mixed-effects model were $R^2m = 0.740$ and $R^2c = 0.904$. A random effect of species could not be included because of limited caudate/anuran combinations and species.

$$ln\ \text{anuran whole-body MeHg}\left(\frac{\text{ng}}{\text{g}}\text{dw}\right) = 0.967 + 0.142 \times ln\ \text{caudate MeHg}\left(\frac{\text{ng}}{\text{g}}\text{dw}\right)$$

Equation S8: Regression equation describing relationships between larval amphibian methylmercury (MeHg; ng/g dry weight [dw]) and Aeshnid-equivalent total mercury (THg; ng/g dw) from a mixed-effects linear model with random effects accounting for variation among sites and species.

$$ln\ \text{larval MeHg}\left(\frac{\text{ng}}{\text{g}}\text{dw}\right) = -0.596 + 0.870 \times ln\ \text{Aeshnid equivalent THg}\left(\frac{\text{ng}}{\text{g}}\text{dw}\right); R^2m = 0.364, R^2c = 0.922$$

Equations S9 and S10: Regression equations describing relationships between larval amphibian methylmercury (MeHg; ng/g dry weight [dw]) and Aeshnid-equivalent total mercury (THg; ng/g dw) from mixed-effects linear models with fixed effects of dragonfly THg concentrations (separate equations for anurans[S9] and caudates [S10]) and random effects accounting for variation among sites and species. These equations are standalone and from subsetted data (anuran and caudate) simplified from the full model (including all data and predictors; see main text).

$$\boldsymbol{S9})\ ln\ \text{anuran MeHg}\left(\frac{\text{ng}}{\text{g}}\text{dw}\right) = 0.337 + 0.596 \times ln\ \text{Aeshnid equivalent THg}\left(\frac{\text{ng}}{\text{g}}\text{dw}\right);\ R^2m = 0.366, R^2c = 0.991$$

$$\boldsymbol{S10})\ ln\ \text{caudate MeHg}\left(\frac{\text{ng}}{\text{g}}\text{dw}\right) = -1.394 + 1.260 \times ln\ \text{Aeshnid equivalent THg}\left(\frac{\text{ng}}{\text{g}}\text{dw}\right); R^2m = 0.771, R^2c = 0.876$$
